# Metabolomic fingerprinting of pig seminal plasma identifies *in vivo* fertility biomarkers

**DOI:** 10.1186/s40104-021-00636-5

**Published:** 2021-11-12

**Authors:** Yentel Mateo-Otero, Pol Fernández-López, Ariadna Delgado-Bermúdez, Pau Nolis, Jordi Roca, Jordi Miró, Isabel Barranco, Marc Yeste

**Affiliations:** 1 Biotechnology of Animal and Human Reproduction (TechnoSperm), Institute of Food and Agricultural Technology, University of Girona, ES-17003 Girona, Spain; 2 Department of Biology, Unit of Cell Biology, Faculty of Sciences, University of Girona, ES-17003 Girona, Spain; 3 Centre d’Estudis Avançats de Blanes (CEAB), Spanish Research Council (CSIC), ES-17300 Girona, Spain; 4 Magnetic Nuclear Resonance Facility, Autonomous University of Barcelona, Bellaterra, ES-08193 Cerdanyola del Vallès, Spain; 5 Department of Animal Medicine and Surgery, Faculty of Veterinary Medicine, University of Murcia, ES-30100 Murcia, Spain; 6 Equine Reproduction Service, Department of Animal Medicine and Surgery, Faculty of Veterinary Medicine, Autonomous University of Barcelona, Bellaterra, ES-08193 Cerdanyola del Vallès, Spain; 7 Department of Veterinary Medical Sciences, University of Bologna, IT-40064 Ozzano dell’Emilia, Bologna, Italy

**Keywords:** Artificial insemination, *in vivo* fertility, Metabolomics, NMR, Pregnancy outcomes, Seminal plasma

## Abstract

**Background:**

Metabolomic approaches, which include the study of low molecular weight molecules, are an emerging -omics technology useful for identification of biomarkers. In this field, nuclear magnetic resonance (NMR) spectroscopy has already been used to uncover (in) fertility biomarkers in the seminal plasma (SP) of several mammalian species. However, NMR studies profiling the porcine SP metabolome to uncover *in vivo* fertility biomarkers are yet to be carried out. Thus, this study aimed to evaluate the putative relationship between SP-metabolites and *in vivo* fertility outcomes. To this end, 24 entire ejaculates (three ejaculates per boar) were collected from artificial insemination (AI)-boars throughout a year (one ejaculate every 4 months). Immediately after collection, ejaculates were centrifuged to obtain SP-samples, which were stored for subsequent metabolomic analysis by NMR spectroscopy. Fertility outcomes from 1525 inseminations were recorded over a year, including farrowing rate, litter size, stillbirths per litter and the duration of pregnancy.

**Results:**

A total of 24 metabolites were identified and quantified in all SP-samples. Receiver operating characteristic (ROC) curve analysis showed that lactate levels in SP had discriminative capacity for farrowing rate (area under the curve [AUC] = 0.764) while carnitine (AUC = 0.847), hypotaurine (AUC = 0.819), sn-glycero-3-phosphocholine (AUC = 0.833), glutamate (AUC = 0.799) and glucose (AUC = 0.750) showed it for litter size. Similarly, citrate (AUC = 0.743), creatine (AUC = 0.812), phenylalanine (AUC = 0.750), tyrosine (AUC = 0.753) and malonate (AUC = 0.868) levels had discriminative capacity for stillbirths per litter; and malonate (AUC = 0.767) and fumarate (AUC = 0.868) levels for gestation length.

**Conclusions:**

The assessment of selected SP-metabolites in ejaculates through NMR spectroscopy could be considered as a promising non-invasive tool to predict *in vivo* fertility outcomes in pigs. Moreover, supplementing AI-doses with specific metabolites should also be envisaged as a way to improve their fertility potential.

**Supplementary Information:**

The online version contains supplementary material available at 10.1186/s40104-021-00636-5.

## Background

Predicting the reproductive potential of sires remains a pending challenge for the livestock industry. This is of particular relevance for the swine sector, whose breeding is mainly based on the use of artificial insemination (AI), an essential tool applied globally to improve reproductive efficiency [1]. Over the last few years, the enhancement of AI-procedures in this species has led to (1) a decrease in the sperm numbers per AI-dose, and (2) a reduction in the number of AI performed per sow, without modifying *in vivo* fertility outcomes [2]. This situation entails that a higher number of AI-doses are elaborated from a single AI-boar and a higher number of sows are inseminated with a single AI-boar, which leads to an increase in the reproductive and economic repercussion of AI-boars on swine farms [2]. Although AI-boars are selected on the basis of their genetic merit and the results obtained by routine sperm analyses (which include sperm concentration, morphology and motility), differences among AI-boars on *in vivo* fertility outcomes are still notable [3, 4]. For this reason, many efforts have been made to uncover biomarkers capable to predict *in vivo* fertility outcomes.

During the past few years, special emphasis has been paid to identify these biomarkers in seminal plasma (SP), a heterogenous fluid secreted by the epididymis and accessory sex glands [5]. This fluid has been poised as a potential source of biomarkers, due to its complex composition and its ability to interact with sperm and the female genital tract, playing a key role in sperm physiology and maternal environment modulation [6–8]. In this sense, high-throughput technologies (including genomics, lipidomics, proteomics, metabolomics and transcriptomics) may hold the key for uncovering reliable fertility biomarkers in SP, since they provide a more in-depth understanding of reproductive processes [9]. In the last decade, many studies conducted in mammalian SP have employed these novel technologies to collect large amounts of data to discover novel fertility biomarkers [10–13].

Metabolomics is the last emerging -omics technology that has become a promising tool to identify biomarkers of (in) fertility [11, 14]. This high-throughput method allows for the study of cells, tissues and biological fluids by evaluating metabolic products, which are the finished outputs of cellular processes [11, 15]. The identification of (in) fertility biomarkers in SP through metabolomics approaches has been extensively reported in several mammalian species, including human [16–19], porcine [20] and bovine [21, 22]. In pigs, Zhang et al. (2021) compared the SP metabolome obtained by ultra-high performance liquid chromatography-quadrupole time-of-flight mass spectrometry between boars with high and low conception rates after AI (< 70 sows inseminated per boar), identifying some SP-metabolites (such as Pro-Asn, Ile-Tyr, and D-Biotin) as potential fertility biomarkers [20]. However, neither the concentration of SP-metabolites, nor the putative relationship between SP-metabolites and other *in vivo* fertility outcomes (e.g. litter size, stillbirths per litter or gestation duration) was reported by these authors.

The aim of this study was to evaluate the relationship between the presence/concentration of SP-metabolites and reproductive performance (including farrowing rate, litter size, stillbirths per litter and duration of pregnancy) of liquid-stored pig semen using Nuclear Magnetic Resonance (NMR) spectroscopy. To achieve this goal, a total of eight AI-boars were included in the study and data from 1,525 inseminations were recorded over a year (> 100 sows inseminated per boar). Using this approach, the present study was able to identify several SP-metabolites able to potentially predict AI outcomes.

## Methods

### Experimental design

A total of 24 entire ejaculates were collected from eight AI-boars (three ejaculates per boar) throughout a year (one ejaculate every 4 months). Immediately after collection, ejaculates were centrifuged to obtain SP-samples, which were stored (− 80 °C) for subsequent metabolomic analysis. Seminal AI-doses (2,400 × 10^6^ spermatozoa in 80 mL) were prepared from these AI-boars and used to inseminate (cervically; two times per estrus) a total of 1,525 weaned multiparous sows (1–7 litters produced) throughout a year. These sows (Landrace and Large White) were housed in different farms in Spain with comparable management conditions. Each boar serviced more than 100 sows.

Fertility outcomes were recorded from each AI-boar included in the study during the same year that SP-samples were collected and AI were performed. Recorded fertility variables were: (1) farrowing rate (percentage of inseminated sows that farrowed), (2) litter size (total number of piglets born per litter), (3) number of stillbirths per litter, and (4) duration of pregnancy (days). These fertility records were corrected for farm-related parameters and sows using the multivariate statistical model described by Broekhuijse et al. [23]. This model allows isolating the direct boar effect on each *in vivo* fertility parameter.

### Boars and ejaculates

All ejaculates were collected from AI-boars housed in a Spanish AI-Center (AIM Iberica, Topigs Norsvin Spain SLU, Calasparra, Murcia, Spain). This center fulfilled the Spanish (ES300130640127, August 2006) and European (ES13RS04P, July 2012) rules in matters of animal health, collection of boar ejaculates and commercialization of AI-doses. As no animal was manipulated by the authors but rather the AI-Center provided AI-doses and fertility data, no permission from an Ethics Committee was required.

The entire ejaculates used in this study were collected from healthy, mature (12 to 36 months), fertile boars from different breeds (Landrace and Large White) using a semi-automatic collection system (Collectis^®^, IMV Technologies, L’Aigle, France). These boars were included in an AI-program and subjected to regular ejaculate collection (twice per week) for producing seminal AI-doses. The entire ejaculates included in this study satisfied the semen quality limits required to produce commercial AI-doses (sperm concentration > 200 × 10^6^ sperm/mL; sperm motility > 70%; sperm with normal morphology > 75%).

Boars were housed in individual pens with controlled temperature (15–25 °C) and light (16 h; natural and artificial). Animals had free access to water and were fed with agricultural feedstuff in agreement with the nutritional requirements of AI-boars.

### Seminal plasma processing and storage

For SP-harvesting, the entire ejaculates were centrifuged (1,500 × *g* for 10 min at room temperature [Rotofix 32A; Hettich Centrifuge UK, Newport Pagnell, Buckinghamshire, England, UK]) twice immediately after ejaculate collection. The resulting second supernatants (SP-samples) were subsequently analyzed (Eclipse E400; Nikon, Tokyo, Japan) to warrant the absence of sperm. Finally, SP-samples were stored in 2-mL cryotubes at − 80 °C (Ultra Low Freezer; Haier Inc., Qingdao, China) until metabolomic profiling was carried out.

### ^1^H NMR analysis

The SP-samples were thawed on ice and one of the aliquots (500 μL) used. Each aliquot was vortexed and centrifuged through 0.5 mL Amicon^®^ Ultra Centrifugal Filters (14,000 × *g* at 4 °C for 90 min) for discarding proteins and cell debris. Then, 100 μL of PBS containing 10% D_2_O with 0.33% of DSS (Merck KgaA, Darmstadt, Germany; pH 7.4) were added to the eluted fractions and transferred into a 5-mm Wilmad^®^ NMR tube (Merck KgaA), where 100 μL of D_2_O was added. Finally, the ^1^H NMR profile was acquired.

### ^1^H NMR spectra

A Bruker 600-MHz AVANCE III NMR spectrometer (Bruker Biospin, Rheinstetten, Germany) operating at a ^1^H frequency of 600.13 MHz and 300 K with a previous equilibration time (10 min) was used to obtain NMR spectra. The 1D-^1^H-nuclear Overhauser effect spectroscopy (1D-NOESY) pulse sequence from the Bruker library was used. The parameters applied were: (1) mixing time: 100 ms (d8); (2) recovery delay: 2 s (d1); (3) 90° pulse: 10.39 μs (p1); (4) spectral width: 7211.539 Hz; (5) spectral size: 32 k; (6) number of scans: 128; and (7) acquisition time: 2.27 s.

### Data processing and analysis

The Chenomx 8.0 profiler software was used for processing and analyzing spectra. This software delivers tools for automatic phase, baseline correction, reference calibration and libraries of metabolites for profiling. The concentration of each metabolite identified in SP was calculated based on DSS concentration (0.216 mmol/L).

### Statistical analysis

All analyses were carried out using R software (version 4.0.2; https://www.r-project.org/). For all analysis, the level of significance was set at *P* ≤ 0.05. Statistical analysis of NMR data was performed in two steps: a) numeric (fertility) vs. numeric (SP-metabolite concentration) variables, and b) categoric (fertility parameter) vs. numeric (SP-metabolite concentration) variables.

First, numerical analysis, namely Pearson correlations, were used to preliminary evaluate the potential linear relationship between SP-metabolite concentration and each fertility parameter.

Onwards, data were split into two different groups for each reproductive parameter (farrowing rates, litter size, stillbirths per litter and pregnancy length). Samples with values lower than the median were classified as negative farrowing rate, decreased litter size and stillbirths per litter, and shorter pregnancy duration; samples with values higher than the median were classified as positive farrowing rate, increased litter size and stillbirths per litter, and longer pregnancy duration. This process was executed for each individual fertility parameter, yielding a specific categorization for each one.

To evaluate potential differences in SP-metabolite concentrations between fertility groups, a Wilcoxon rank sum test (equivalent to Mann-Whitney U test) was performed. As opposed to *t*-test, Wilcoxon does not assume normal distribution of samples, which did not occur in some cases. A multivariate analysis was also carried out to evaluate putative inter-metabolite relationships and patterns that could predict fertility outcomes. In this sense, a Bayesian logistic regression was used, treating the groups above the median as success (1) and those below the median as failure (0). An individual model was run for each of the fertility parameters considering all the metabolites as potential predictors, using the R package ‘rstanarm’ (R package version 2.21.1; [24]), with non-informative prior distributions, high resolution sampling of the posterior distribution (adapt_delta = 0.99) and 4,000 iterations. The remaining parameters of the models were left by default. The Bayesian framework was selected over the classical frequentist one because of the structure of data. Usually, with a higher number of features (or predictors) than samples, as in the present dataset, models tend to get overfitted. While classical regression models rely on confidence intervals to estimate their reliability, the Bayesian framework estimates the whole posterior (the approximately ‘real’) distribution and allows quantifying the uncertainty of coefficients and predictions accordingly. This methodology is particularly of interest not only for having a good sense of how accurate predictions are, but also for improving the models as more information about data becomes available (e.g., knowledge about the mean or the range of the ‘true’ distribution of the predictors).

As a last step to assess the predictability of the different fertility parameters, two additional analyses were performed. A sparse partial least square discriminant analysis (sPLS-DA) model was run separately for each of the parameters, using the ‘mixOmics’ R package [25]. Similar to principal components analysis (PCA), this method is useful for identifying key features in the dataset. However, while PCA relies on maximizing the variance of the features in the principal components, sPLS-DA maximizes their covariance. Metabolites that were relevant in the sPLS-DA analysis and/or exhibited differences between groups were further tested in a Receiver Operating Characteristic (ROC) curve, using the ‘pROC’ package for R [26]. This method allows for further validation of the predictive performance of metabolites and provides a ‘cut-off’ or threshold value to discriminate (or predict) sample fertility (‘high’ or ‘low’). Results are expressed as the area under the curve (AUC). The discriminant relevance was measured by the following AUC ranges: 0.0–0.5 = no discriminant value, 0.5–0.6 fail discriminant value, 0.6–0.7 poor discriminant value, 0.7–0.8 fair discriminant value, 0.8–0.9 good discriminant value, and 0.9–1 excellent discriminant value.

## Results

### Metabolite profile of pig SP

The ^1^H-NMR profile allowed the identification and quantification of a total of 24 metabolites in pig SP-samples **(**see Supplementary Fig. 1). The identified metabolites were categorized in: i) amino acids (*n* = 7; alanine, glutamate, isoleucine, leucine, phenylalanine, tyrosine and valine); ii) alcohols (*n* = 2; ethanol and methanol); iii) saccharides (*n* = 1; glucose); iv) salts (*n* = 7; acetate, benzoate, citrate, formate, fumarate, lactate and malonate); and v) other organic compounds (*n* = 5; carnitine, creatine, creatine-phosphate, hypotaurine, myo-inositol, sn-glycero-3-phosphocholine and trimethylamine N-oxide).

### Association between SP-metabolites and AI outcomes

**Fig. 1:**
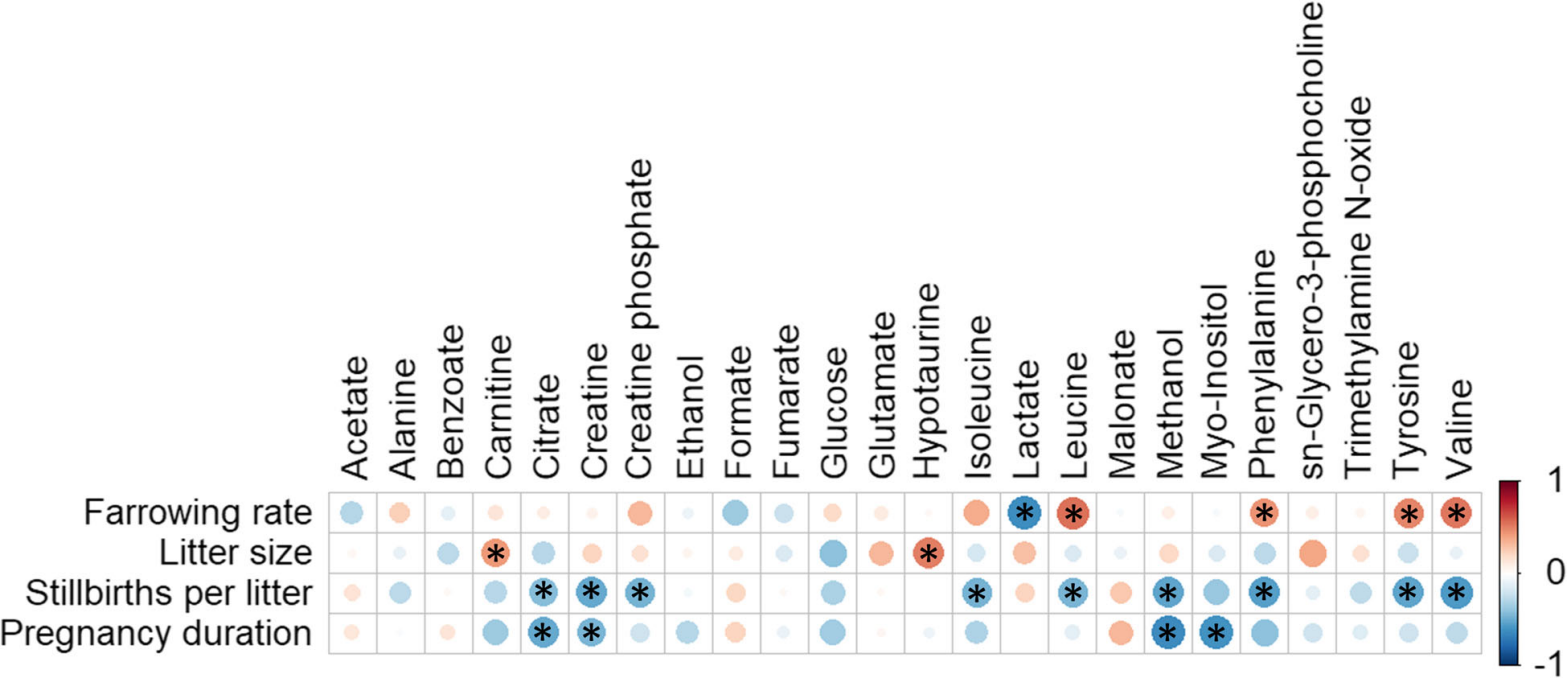
Correlations between pig seminal plasma-metabolites and *in vivo* fertility outcomes (farrowing rates, litter size, stillbirths per litter and gestion duration). Seminal plasma samples from entire ejaculates (24 ejaculates) of eight artificial insemination-boars (three ejaculates per boar) were used. Fertility parameters were recorded from 1,525 inseminations. The colour saturation of red to blue represents the correlation coefficients (R) between metabolites, from 1 to − 1, respectively. Significant correlations (*P* < 0.05) are marked with *

Correlations between the concentration of SP-metabolites and *in vivo* fertility parameters were calculated **(**Fig. 1**)**. Five SP-metabolites were found to be correlated (*P* < 0.05) with farrowing rate: lactate (R = − 0.62), leucine (R = 0.55), phenylalanine (R = 0.45), tyrosine (R = 0.49) and valine (R = 0.53). Moreover, two SP-metabolites were positively correlated (*P* < 0.05) with litter size: carnitine (R = 0.42) and hypotaurine (R = 0.51). Additionally, the number of stillbirths per litter was negatively correlated (*P* < 0.05) with nine SP-metabolites: citrate (R = − 0.42), creatine (R = − 0.51), creatine phosphate (R = − 0.46), isoleucine (R = − 0.47), leucine (R = − 0.46), methanol (R = − 0.53), phenylalanine (R = − 0.54), tyrosine (R = − 0.52) and valine (R = 0.57). Finally, the duration of gestation was negatively correlated (*P* < 0.05) with four SP-metabolites: citrate (R = − 0.51), creatine (R = − 0.45), methanol (R = − 0.63) and myo-inositol (R = − 0.59).

Bayesian multiple logistic regression analyses were carried out with the aim (i) to develop a potential predictive model, (ii) to quantify the relative contribution of each SP-metabolite to each *in vivo* fertility parameter, and (iii) to identify specific metabolite patterns that may have an influence on *in vivo* fertility parameters. However, no model showed a clear relationship with any of the reproductive outcomes **(**Supplementary Fig. 2A-D).

### Relationship between SP-metabolites and farrowing rate

Boars were classified into two groups based on their farrowing rate deviation from the median: negative farrowing rate deviation (ranging from − 2.80 to − 1.60; *n* = 4) and positive farrowing rate deviation (ranging from 2.82 to 7.54; *n* = 4). Only the concentration of lactate in SP differed (*P* < 0.05) between groups, showing higher levels in SP-samples from boars with negative farrowing rate deviation (median ± SD; 1.90 mmol/L ± 0.508) compared to those with positive farrowing rate deviation (median ± SD; 1.22 mmol/L ± 0.585).

**Fig. 2:**
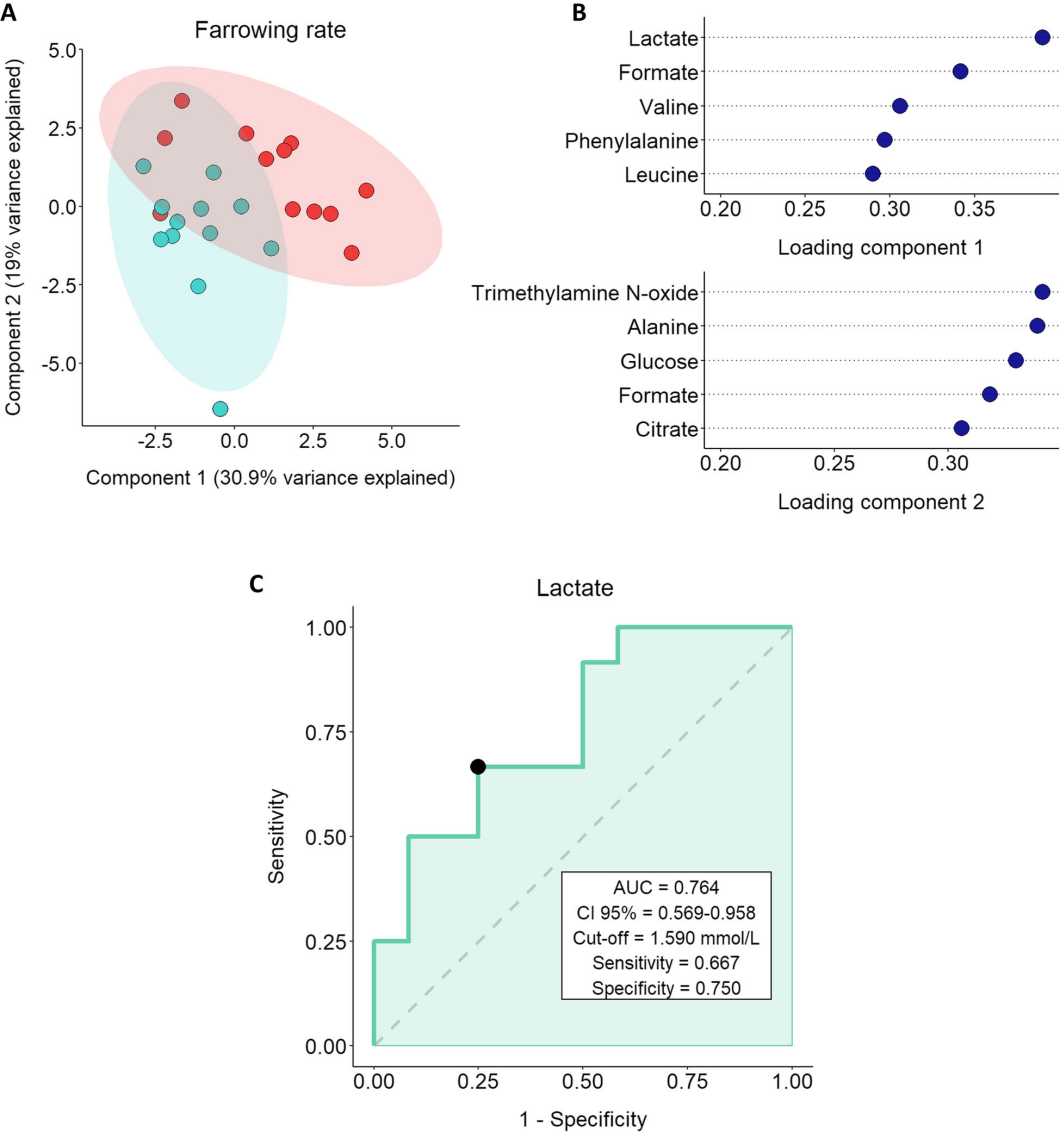
**A** sPLS-DA analysis for farrowing rates deviation showing sample distribution in component 1 and component 2. Seminal plasma samples from entire ejaculates (24 ejaculates) of eight artificial insemination-boars (three ejaculates per boar) were used. Fertility parameters were recorded from 1525 inseminations. The colored areas represent the 95% confidence interval. Boars classified with positive farrowing rate deviation (ranging from 2.82 to 7.54; *n* = 4) are represented in red and those with negative farrowing rate deviation (ranging from − 2.80 to − 1.60; *n* = 4) are shown in blue. Each dot symbolizes an ejaculate. **B** Loading plot for components 1 and 2 for the sPLS-DA model. Variables are ranked by the absolute values of their loadings. **C** Receiver operating characteristic (ROC) curve analysis for lactate concentration in seminal plasma and farrowing rate deviation. Seminal plasma samples from entire ejaculates (24 ejaculates) of eight artificial insemination-boars (three ejaculates per boar) were used. Fertility parameters were recorded from 1,525 inseminations. The plot shows the ability of a given metabolite to discriminate farrowing rate of semen doses. AUC: area under the curve; CI: confidence interval

The sPLS-DA analysis was carried out to select the most predictive or discriminant features in the dataset to classify samples [27]. The sPLS-DA analysis for farrowing rates deviation using the first two components explained 49.9% of the total variance of the sample **(**Fig. 2A**)**. The resulting plot showed two different groups: SP-samples from boars exhibiting negative farrowing rate deviation (blue) were mainly discriminated by the second component, whereas SP-samples from boars classified as positive farrowing rate deviation (red) were separated by the first component. The loadings plot, which shows the most relevant variable for a given component, revealed that whereas lactate and formate were the most important variables for the first component, trimethylamine N-oxide and alanine were the most relevant for the second one **(**Fig. 2B**)**. ROC curve analysis indicated that only lactate was able to predict farrowing rate deviation (*P* < 0.05; Fig. 2C). Specifically, lactate showed a fair discriminant value with an AUC of 0.764.

### Relationship between SP-metabolites and litter size

Boars were classified into two groups depending on their litter size deviation from the median: reduced litter size (ranging from − 0.40 to 0.02; *n* = 4) and increased litter size (ranging from 0.11 to 0.52; *n* = 4) deviation. Concentrations of carnitine, hypotaurine, sn-glycero-3-phosphocholine and glutamate in SP differed (*P* < 0.05) between groups, displaying higher levels in SP-samples from boars with increased litter size deviation than in SP-samples from boars with reduced litter size deviation (median ± SD; for carnitine: 0.82 mmol/L ± 0.223 vs. 0.43 mmol/L ± 0.244; for glutamate: 1.71 mmol/L ± 0.437 vs. 1.33 mmol/L ± 0.607; for hypotaurine: 2.85 mmol/L ± 0.604 vs. 1.77 mmol/L ± 0.813; for sn-glycero-3-phosphocholine: 6.45 mmol/L ± 1.373 vs. 4.69 mmol/L ± 1.932, respectively). Concentration of glucose in SP also differed (*P* < 0.05) between groups, showing the opposite pattern to the aforementioned metabolites; indeed, SP-samples from boars with increased litter size deviation (median ± SD; 0.24 mmol/L ± 0.273) exhibited lower glucose concentration than those with decreased litter size deviation (median ± SD; 0.79 mmol/L ± 0.245).

**Fig. 3:**
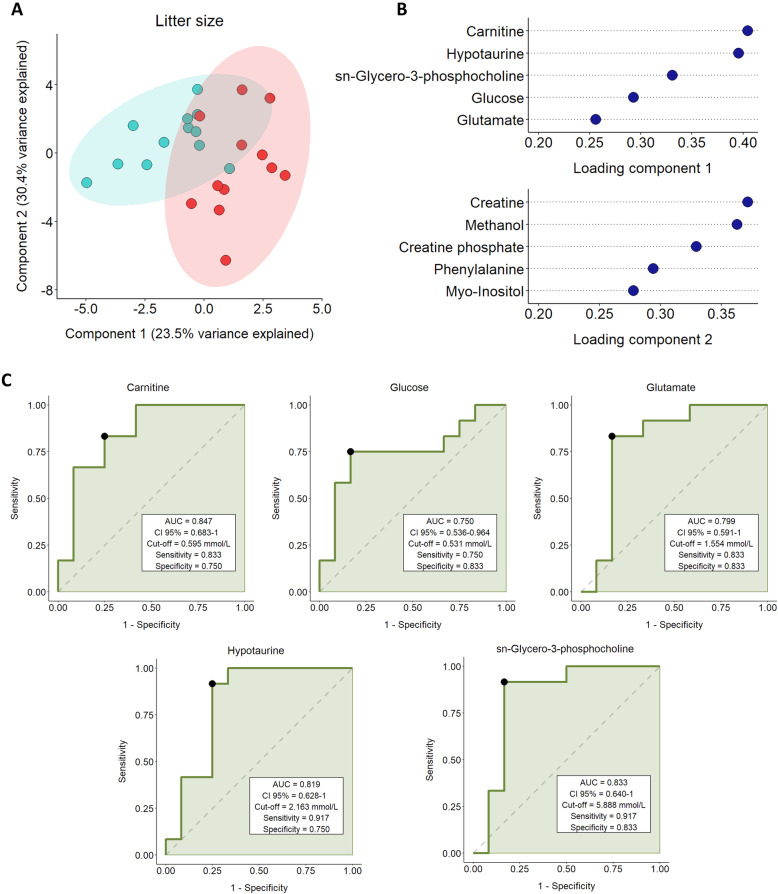
**A** sPLS-DA analysis for litter size deviation showing sample distribution in component 1 and component 2**.** Seminal plasma samples from entire ejaculates (24 ejaculates) of eight artificial insemination-boars (three ejaculates per boar) were used. Fertility parameters were recorded from 1525 inseminations. The colored areas represent the 95% confidence interval. Boars with decreased litter size deviation (ranging from − 0.40 to 0.02; *n *= 4) are represented in blue and those with increased litter size deviation (ranging from 0.11 to 0.52; *n* = 4) are shown in red. Each dot symbolizes an ejaculate. **B** Loading plot for components 1 and 2 for the sPLS-DA model. Variables are ranked by the absolute values of their loadings. **C** Receiver operating characteristic (ROC) curve analysis for carnitine, glucose, sn-glycero-3-phosphocholine, glutamate and hypotaurine concentrations in seminal plasma and litter size deviation. Seminal plasma samples from entire ejaculates (24 ejaculates) of eight artificial insemination-boars (three ejaculates per boar) were used. Fertility parameters were recorded from 1,525 inseminations. The plot shows the ability of the metabolites to discriminate litter size of semen doses. AUC: area under the curve; CI: confidence interval

The sPLS-DA analysis **(**Fig. 3A**)** showed that the first two components explained 53.9% of the total variance of the sample. The plot showed two different groups; SP-samples from boars classified with increased litter size deviation (red) were mainly influenced by the second component, whereas SP-samples from boars classified with decreased litter size deviation (blue) were mainly affected by the first component. The loadings plot revealed that while carnitine, hypotaurine, sn-glycero-3-phosphocholine and glucose strongly influenced the first component, glutamate and methanol had that effect on the second component **(**Fig. 3B**)**. ROC curve analysis showed that all SP-metabolites identified as relevant by the loadings plot had a significant AUC (*P* < 0.05; Fig. 3C). Specifically, carnitine showed the highest AUC of 0.840, hypotaurine displayed an AUC of 0.819, sn-glycero-3-phosphocholine showed an AUC of 0.833, glucose exhibited an AUC of 0.750, and glutamate had an AUC of 0.799. Thus, the ROC curve showed that while carnitine, hypotaurine, sn-glycero-3-phosphocholine and glutamate exhibited a good discriminant value for predicting litter size due to their high AUC (ranging 0.8–0.9), glucose had a fair discriminant predictive value (ranging 0.6–0.7) for litter size.

### Relationship between SP-metabolites and the number of stillbirths per litter

Boars were categorized into two groups depending on their stillbirths per litter deviation from the median: decreased stillbirths per litter deviation (ranging from − 0.10 to 0.02; *n* = 4) and increased stillbirths per litter deviation (ranging from 0.05 to 0.14; *n* = 4). Concentrations of citrate, creatine, phenylalanine and tyrosine in SP differed (*P* < 0.05) between groups: the SP-samples from boars with decreased stillbirths per litter deviation showing higher concentrations than those from boars with increased stillbirths per litter (median ± SD; for citrate: 7.10 mmol/L ± 1.738 vs. 4.95 mmol/L ± 2.192; for creatine: 0.54 mmol/L ± 0.158 vs. 0.31 mmol/L ± 0.156; for phenylalanine: 0.03 mmol/L ± 0.011 vs. 0.02 mmol/L ± 0.012; for tyrosine: 0.03 mmol/L ± 0.017 vs. 0.02 mmol/L ± 0.016, respectively). In an opposite manner, malonate was found to be higher (*P* < 0.05) in SP-samples from boars with increased stillbirths per litter (median ± SD; 0.16 mmol/L ± 0.064) than in those from boars with decreased stillbirths per litter (median ± SD; 0.09 mmol/L ± 0.054).

**Fig. 4:**
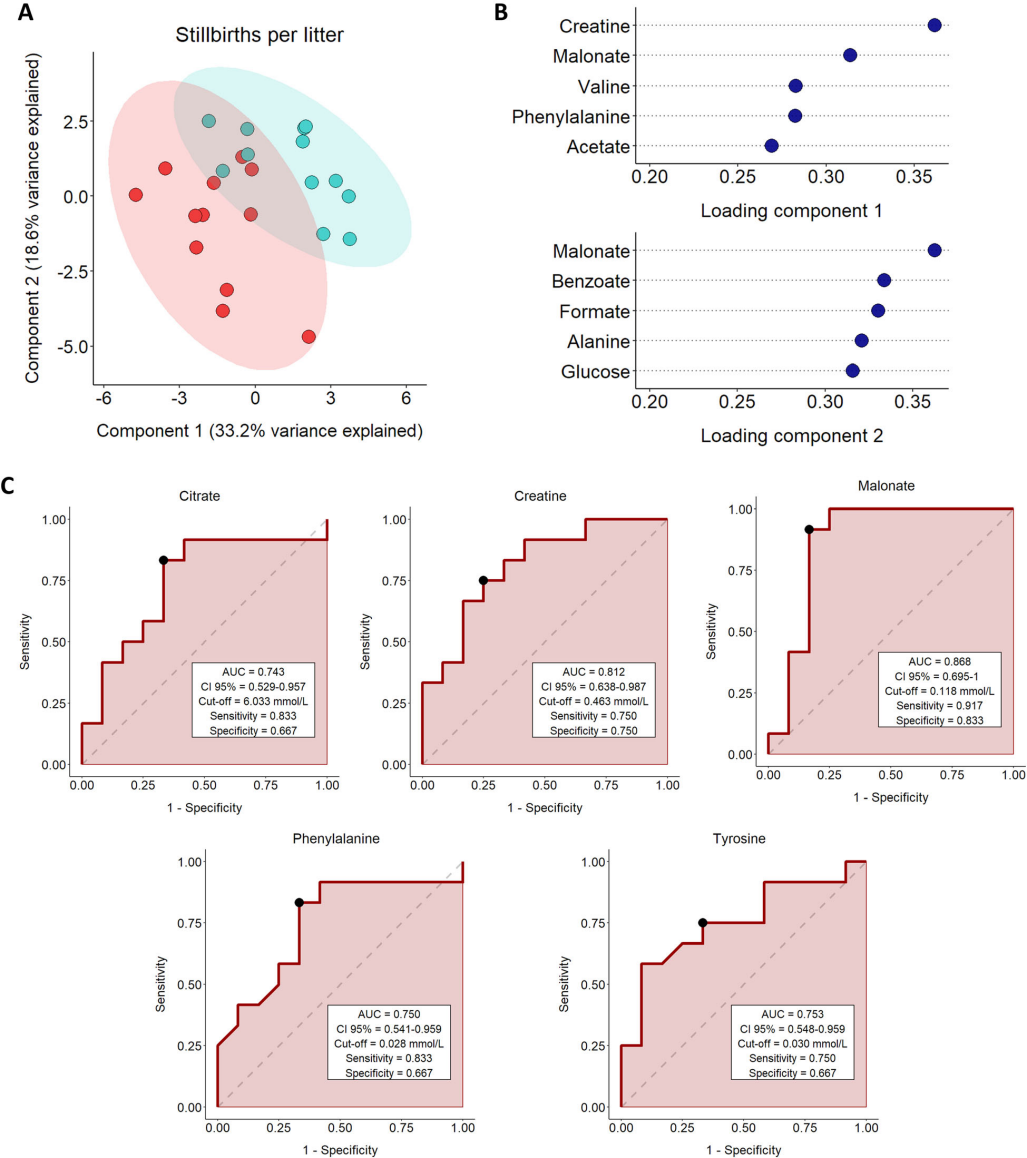
**A** sPLS-DA analysis for the number stillbirths per litter deviation showing sample distribution in component 1 and component 2. Seminal plasma samples from entire ejaculates (24 ejaculates) of eight artificial insemination-boars (three ejaculates per boar) were used. Fertility parameters were recorded from 1,525 inseminations. The colored areas represent the 95% confidence interval. Boars with decreased stillbirths per litter deviation (ranging from − 0.10 to 0.02; *n* = 4) are represented in blue and those with increased stillbirths per litter deviation (ranging from 0.05 to 0.14; *n *= 4) are shown in red. Each dot symbolizes an ejaculate. **B** Loading plot for components 1 and 2 for the sPLS-DA model. Variables are ranked by the absolute values of their loadings. **C** Receiver operating characteristic (ROC) curve analysis for citrate, creatine, phenylalanine, tyrosine and malonate and stillbirths per litter. They show the ability of metabolites to discriminate the number of stillbirths per litter after artificial insemination with semen doses. AUC: area under the curve; CI: confidence interval

Regarding the sPLS-DA analysis **(**Fig. 4A**)**, the first two components were found to explain 51.8% of the total variance. Moreover, two different groups were observed: while SP-samples from boars with decreased deviation in the number of stillbirths per litter (blue) were mainly influenced by both components, SP-samples from boars with increased deviation (red) were influenced by the second component. The loadings plot revealed that the first component was mainly influenced by creatine and malonate, and the second component by malonate, benzoate and formate **(**Fig. 4B**)**. ROC curve analysis showed that the AUC was significant (*P* < 0.05; Fig. 4C) for citrate, creatine, malonate, phenylalanine and tyrosine. Specifically, citrate exhibited an AUC of 0.743, creatine displayed an AUC of 0.812, malonate showed an AUC of 0.868, phenylalanine displayed an AUC of 0.750, and tyrosine showed an AUC of 0.753. Therefore, the ROC curve revealed that while creatine and malonate had a good discriminant value for predicting stillbirths per litter (as their AUC ranged from 0.8 to 0.9), citrate, creatine and tyrosine displayed a fair discriminant strength (as their AUC ranged from 0.7 to 0.8).

### Relationship between SP-metabolites and duration of gestation

Boars were classified into two groups depending on the deviation of gestation duration from the median, i.e. shorter gestation duration (ranging from − 0.85 to 0.03; *n* = 4) and longer gestation duration (ranging from 0.10 to 0.52; *n* = 4) deviation. Concentration of malonate in SP differed (*P* < 0.05) between groups, showing higher levels in SP-samples from boars with longer gestation duration deviation (median ± SD; 0.16 mmol/L ± 0.072) compared to those from boars with shorter gestation duration deviation (median ± SD; 0.09 mmol/L ± 0.030). On the contrary, fumarate exhibited higher levels (*P* < 0.05) in SP-samples from boars with shorter gestation duration (median ± SD; 0.01 mmol/L ± 0.002) than in those from boars with longer gestation duration (median ± SD; 0.004 mmol/L ± 0.002).

**Fig. 5:**
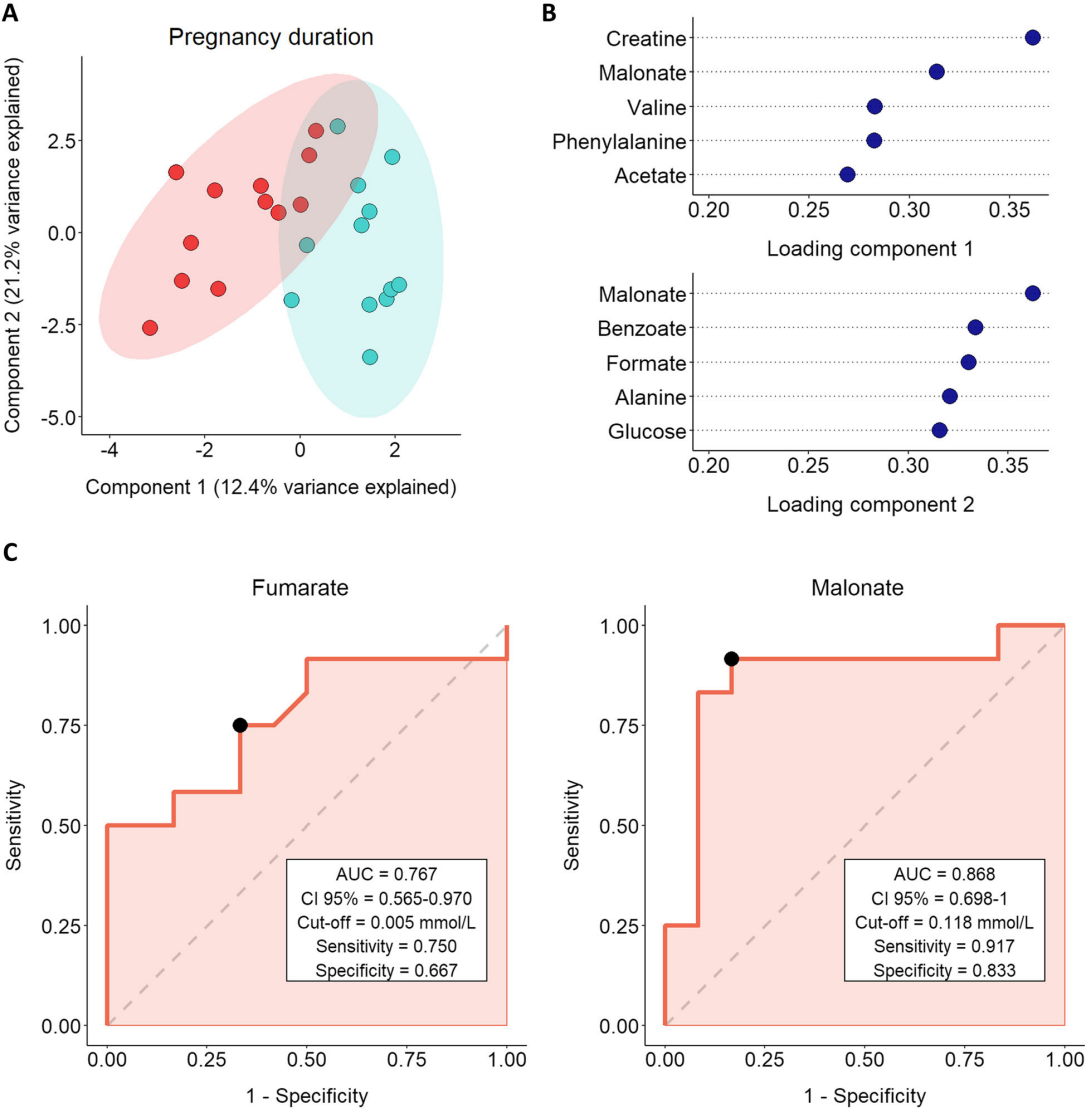
**A** sPLS-DA analysis for gestation duration deviation showing sample distribution in component 1 and component 2. Seminal plasma samples from entire ejaculates (24 ejaculates) of eight artificial insemination-boars (three ejaculates per boar) were used. Fertility parameters were recorded from 1,525 inseminations. The colored areas represent the 95% confidence interval. Boars with longer gestation duration deviation (ranging from 0.10 to 0.52; *n* = 4) are represented in red and those with shorter gestation duration deviation (ranging from − 0.85 to 0.03; *n* = 4) are shown in blue. Each dot symbolizes an ejaculate. **B** Loading plot for components 1 and 2 for the sPLS-DA model. Variables are ranked by the absolute values of their loadings. **C** Receiver operating characteristic (ROC) curve analysis for malonate and fumarate and pregnancy duration. They show the ability of metabolites to discriminate the number of stillbirths per litter after artificial insemination with semen doses. AUC: area under the curve; CI: confidence interval

sPLS-DA analysis for gestation duration showed that the first two components explained 33.6% of the total variance **(**Fig. 5A**)**. Again, two different groups were identified: while SP-samples from boars classified with a shorter deviation in the gestation duration (blue) were mainly affected by the second component, SP-samples from boars with longer deviation (red) were equally influenced by both components. The loadings plot revealed that the first component was strongly influenced by malonate, and the second component by glutamate, sn-glycero-3-phosphocholine and carnitine **(**Fig. 5B**)**. For these SP-metabolites, both malonate and fumarate showed a significant ROC curve (*P* < 0.05; Fig. 5C). Specifically, malonate exhibited an AUC of 0.868 and fumarate showed an AUC of 0.767. Considering these results, the ROC curve revealed that while malonate had a good discriminant value for predicting gestation duration, fumarate showed a fair discriminant predictive value for this parameter.

## Discussion

In the last years, metabolite identification and quantification for male infertility assessment has become an emerging area of research [9, 10, 28]. In this field, NMR spectroscopy is one of the three most common analytical methods for metabolite profiling [28]. The present report evaluated the potential relationship between SP-metabolite concentrations and AI outcomes in pigs using NMR approaches, figuring out which SP-metabolites could be used as *in vivo* fertility biomarkers. Specifically, this study demonstrated that: i) the concentration of SP-lactate was related to farrowing rate; ii) concentrations of carnitine, hypotaurine, sn-glycero-3-phosphocholine glutamate and glucose in SP were associated with litter size; iii) concentrations of citrate, creatine, malonate, phenylalanine and tyrosine in SP were related to the number of stillbirths per litter; and iv) concentrations of malonate and fumarate in SP were associated to gestation duration.

In accordance with our previous report [29], this study identified and quantified a total of 24 SP-metabolites. In addition, the results of the present work showed that several SP-metabolites were related to specific *in vivo* fertility parameters. However, since all the relationships were found to be moderate (as Pearson correlation coefficients were lower than 0.6) and no SP-metabolite pattern for specific fertility parameters was observed using Bayesian multiple logistic models, sPLS-DA and ROC analysis were run. Using these statistic tools, potential biomarkers for all the assessed reproductive performance variables were identified.

The results of the present study evidenced that lactate was the only SP-metabolite related to farrowing rate. These results differ from those reported by Zhang et al. who found that several amino acids and D-biotin in SP were related with conception rates in pigs [20]. Differences in (1) the analytical method (mass spectrometry vs. NMR) and in (2) the analysis of fertility records, since the work of Zhang et al. did not take other parameters that could influence conception rates (sows, farms …) into consideration [20], may contribute to explain the divergent results between both studies. In the present work, the highest lactate concentrations were found in SP-samples from boars classified with negative farrowing rate deviation. It is well known that lactate is one of the main non-monosaccharide substrates for sperm in bulls [30], stallions [31], men [32] and boars [32, 33]. Non-oxidative metabolism of pig sperm has been shown to consume lactate [34], which is transformed into pyruvate through lactate dehydrogenase to produce ATP [35]. Considering these findings, one could assume that sperm from boars classified with positive farrowing rate deviation could better metabolize lactate for energy production, thereby leading to lower SP-lactate concentration, as confirmed by the present study. However, it is worth mentioning that these results are not in agreement with previous findings reported in cattle, in which the highest lactate levels were found in the SP of high fertility bulls [30], and in men, as infertile patients had lower SP-lactate levels than fertile controls [16, 18, 36, 37]. These differences could be attributed to: (1) different metabolic sperm strategies, as while pig sperm can use lactate as an energy source, the rate between oxidative phosphorylation and glycolysis is higher in bovine sperm [38]; and/or (2) differences in SP composition as a result of differences in mating strategies between species [39, 40].

Regarding the litter size, the present study showed that concentrations of glucose, carnitine, hypotaurine, sn-glycero-3-phosphocholine and glutamate were related with this fertility parameter. Interestingly, the ROC curve revealed that these SP-metabolites had a discriminating ability to predict the litter size, so that all the four could be considered as promising biomarkers for this AI outcome.

It is well known that glucose is one of the main monosaccharides used by mammalian sperm to produce energy [32, 41]. The present study reported that boars classified with an increased litter size deviation exhibited lower glucose concentrations in their SP than those with a decreased litter size deviation. Similar findings were reported in humans, in which men with idiopathic infertility had higher glucose levels in their SP compared to healthy individuals [17]. The most feasible explanation for such findings would be that sperm from boars with a decreased litter size would consume less glucose from SP; thus, glucose would be extracellularly accumulated. This hypothesis would be in agreement with the existing literature, as the supplementation of semen extenders with glucose has been reported to increase sperm motility and ATP concentration in humans [42]. Taken these data together, it could be suggested that low levels of glucose in SP are beneficial for both sperm physiology and reproductive performance.

Carnitine is an antioxidant that has been widely demonstrated to be involved in mammalian sperm motility [43, 44]. Moreover, a protective role of this antioxidant on DNA and plasma membrane oxidation damage in humans [44, 45] and pigs [46] has also been reported. In addition, dietary carnitine supplementation in boars has been proved to improve sperm quality parameters [47, 48]. In agreement with these studies, the results reported herein indicate that boars with an increased litter size deviation exhibit the highest SP-concentration of this metabolite. These results are in accordance with the study of Zöpfgen et al., who found that infertile men had lower SP-levels of carnitine than their fertile counterparts [49]. Nevertheless, this result, together with the aforementioned findings, open the possibility of using the measurement of carnitine in SP as a potential litter size biomarker.

Hypotaurine is an antioxidant present in human SP and sperm [50, 51]. The present study found a positive influence of SP-hypotaurine on litter size. This relationship could be driven by both an effect on sperm and/or oocyte fertilization. With regard to sperm, the addition of hypotaurine to cryopreservation media has been reported to exert a positive effect on sperm quality and functionality parameters in sheep [52] and humans [51, 53]. In addition, sperm from bulls with high fertility records also have high hypotaurine levels [54]. While, considering all this evidence, one could surmise that SP-hypotaurine has a positive impact on pig sperm physiology, further studies are required to confirm this hypothesis. On the other hand, supplementation of *in vitro* culture media with hypotaurine increases embryo cleavage and, in consequence, embryo development in bovine [55] and improves the intracellular oxidative status of pre-implantational porcine embryos [56]. Thus, SP-hypotaurine could also affect early embryo development stages, thus increasing litter size.

Glutamate is an amino acid involved in cellular energy production and in the synthesis of many other amino acids and nucleotides [18]. Low levels of SP-glutamate have been related to several forms of infertility in humans [18, 37]. In agreement with these results, the present study found that higher levels of SP-glutamate were related to increased litter size deviation. Based on these data, the effect of glutamate on AI outcomes could be driven by its repercussion on sperm, as equine intracellular glutamate has been proposed: i) to contribute to sperm functionality through its metabolization via non-canonical pathways; and ii) to be exchanged for extracellular cysteine to produce reduced glutathione [57]. Nonetheless, before could glutamate be used as a litter size biomarker, the aforementioned hypothesis should be tested in the pig.

Finally, sn-glycero-3-phosphocholine, which is involved in glycerophospholipid metabolism, has been reported to play a vital role in sperm capacitation and acrosome reaction in rats [58]. The results of the present study showed a positive relationship between sn-glycero-3-phosphocholine concentration in SP and high litter size. These results seem to agree with previous studies performed in other species, in which infertile men were observed to exhibit lower sn-glycero-3-phosphocholine levels in their SP compared to their fertile counterparts [59]. In addition, it has been reported that rat sperm head accumulates lipid metabolites as a result of sn-glycero-3-phosphocholine metabolism during acrosome reaction, which could have an involvement in sperm-oocyte interaction and even in gamete fusion [58]. Considering all these findings, further research addressing the specific role played by SP-sn-glycero-3-phosphocholine in pig fertility is warranted.

The present study also evaluated the relationship between SP-metabolites and stillbirths per litter. Citrate, creatine, phenylalanine and tyrosine were observed to be promising biomarkers for stillbirths per litter due to their ROC curves. In this sense, citrate is involved in the Krebs cycle, which is the most relevant metabolic pathway for energy production [60]. The results of the present study revealed that high SP concentrations of this metabolite were related to a low number of stillbirths per litter. These results came as a surprise considering that low levels of SP-citrate have been observed in high-fertility bulls [21] and SP-citrate has been widely proposed as a biomarker for different human infertility forms [18, 36, 61]. Considering the opposite trend of the results presented herein, the exact mechanism through which SP-citrate could positively influence AI outcomes needs to be clarified in future studies.

Creatine is involved in the regulation of ATP and both the supplementation of *in vitro* fertilization medium with creatine [62] and the presence of this metabolite in SP [63] have been reported to influence sperm physiology in terms of motility and viability in humans [62, 63]. Interestingly, the present study found that high SP-creatine concentration was associated with decreased stillbirths per litter deviation. This result may be explained by the fact that creatine has been found to enhance fertilization and promote blastocyst and normal embryo development [62]. Consequently, although this should be further confirmed, it could be posited that high SP-creatine has a positive effect on both gametes, thus improving AI outcomes and decreasing the number of stillbirths per litter.

Phenylalanine and tyrosine, amino acids involved in the same metabolic pathway [64], were found to be higher in SP-samples from boars with decreased stillbirths per litter deviation. While, to the best of our knowledge, no information about the effect of tyrosine on sperm physiology has been reported, phenylalanine is known to stimulate the ability of human sperm to capacitate and undergo acrosomal exocytosis [65]. In cattle, phenylalanine levels in SP are positively related to post-thaw sperm viability, suggesting that this amino acid could be involved in oxidoreductase and oxidant reactions [66]. Interestingly, SP-tyrosine has also been found to contribute to the total antioxidant capacity of SP [67]. If these findings were confirmed in pigs, concentrations of phenylalanine and tyrosine in SP would also appear as exerting a beneficial effect upon sperm through regulation of reactive oxygen species (ROS) and could be used to predict fertility outcomes in porcine.

Finally, the relationship between gestation duration and concentration of SP-metabolites was also investigated, and whereas malonate showed higher levels in SP-samples from boars with longer gestation duration, fumarate exhibited lower levels in that group. However, further studies for fumarate validation should be conducted, as no information regarding the effect of this metabolite on sperm physiology or fertilizing ability has been published. On the other hand, malonate may have a double predictive value for both stillbirths per litter size and the estimation of gestation duration. Malonate is an intermediate metabolite of the Krebs cycle that inhibits ROS production via competition for succinate dehydrogenase [68, 69]. The present study identified a positive relationship between SP-malonate levels and both stillbirths per litter size and gestation duration. A similar negative influence of SP-malonate has also been found in humans, as infertile patients exhibited higher malonate levels than fertile controls [70]. On the other hand, malonate can act as protein post-translational modification [71]. Based on these findings, while no studies have been conducted to evaluate the influence of malonate on sperm physiology, it could be posited that a high SP-malonate concentration could: i) modify key proteins involved in gamete interaction or even embryo development, or ii) inhibit the Krebs cycle. In any case, the fact that malonate relates to two distinct *in vivo* fertility parameters reinforces its potential value as a predictor of AI outcomes in pig SP.

As aforementioned, the differences found between the results of the present research and those reported in other metabolomic studies conducted in pigs or in other species may be due to several factors: i) differences in the sensitivity of the metabolomic approaches; ii) variations in the preparation of samples; iii) the species-specific role of seminal metabolites in fertility; and iv) the use of non-comparable fertility parameters. For this reason, although -omics approaches are powerful tools, they should be used as a first steppingstone in the research of (in-)fertility biomarkers [10, 29, 72]. In effect, while the main strength of the present work is that a set of SP-metabolites has been proposed to predict AI outcomes, they should all be further validated using a higher number of individuals and other approaches to overcome the intrinsic limitations of-omics approaches. Following this, the measurement of metabolites in SP could be potentially used as an accurate fertility test to select boars before they are included in an AI-program. Moreover, future research needs to be conducted to assess i) the specific role of each SP-metabolite in male fertility, and ii) whether supplementing AI-extenders with specific metabolites can improve the fertility potential of semen doses.

## Conclusions

The metabolite profiling of pig SP using NMR spectroscopy allowed the identification and quantification of 24 metabolites. The results evidenced that 13 of these metabolites were related with AI outcomes, pointing out to putative *in vivo* fertility biomarkers. Specifically, lactate could be used as a farrowing rate indicator; carnitine, hypotaurine, sn-glycero-3-phosphocholine, glutamate and glucose could predict litter size; citrate, creatine, phenylalanine, tyrosine and malonate would be biomarkers for the number of stillbirths per litter; and, finally, malonate and fumarate would anticipate the duration of gestation.

## Supplementary Information

**Additional file 1 Supplementary Fig. 1** H-NMR (noesygppr1d) profile (600 MHz) from 0 to 8 ppm of pig seminal plasma.

**Additional file 2 Supplementary Fig. 2 (A-D).** Bayesian multiple logistic regression models for all the *in vivo* fertility parameters. The distribution of the coefficients (X axis) is depicted for each metabolite (Y axis). The coefficient distributions depict their effect on the model, as well as their associated uncertainties (credible intervals). Thus, changes in one unit on the coefficient value has a multiplicative effect on the log-odds of the prediction, equal to the value of the coefficient. Blue lines represent the 95% credible intervals, boxes show the 50% credible intervals, and dots are the distribution median.

## Data Availability

All data generated or analyzed during this study are included in this published article (and its supplementary information files).

## Abbreviations

AI: Artificial insemination
AUC: Area under the curve
NMR: Nuclear magnetic resonance
PCA: Principal components analysis
ROC: Receiver operating characteristic
ROS: Reactive oxygen species
SP: Seminal plasma
sPLS-DA: Sparse partial least square discriminant analysis
