# Supplementary figures and images for: Metabolomic fingerprinting of pig seminal plasma identifies in vivo fertility biomarkers
Source: J Anim Sci Biotechnol. 2021 Nov 12;12:113. doi: 10.1186/s40104-021-00636-5 (PMC8588628; doi:10.1186/s40104-021-00636-5)

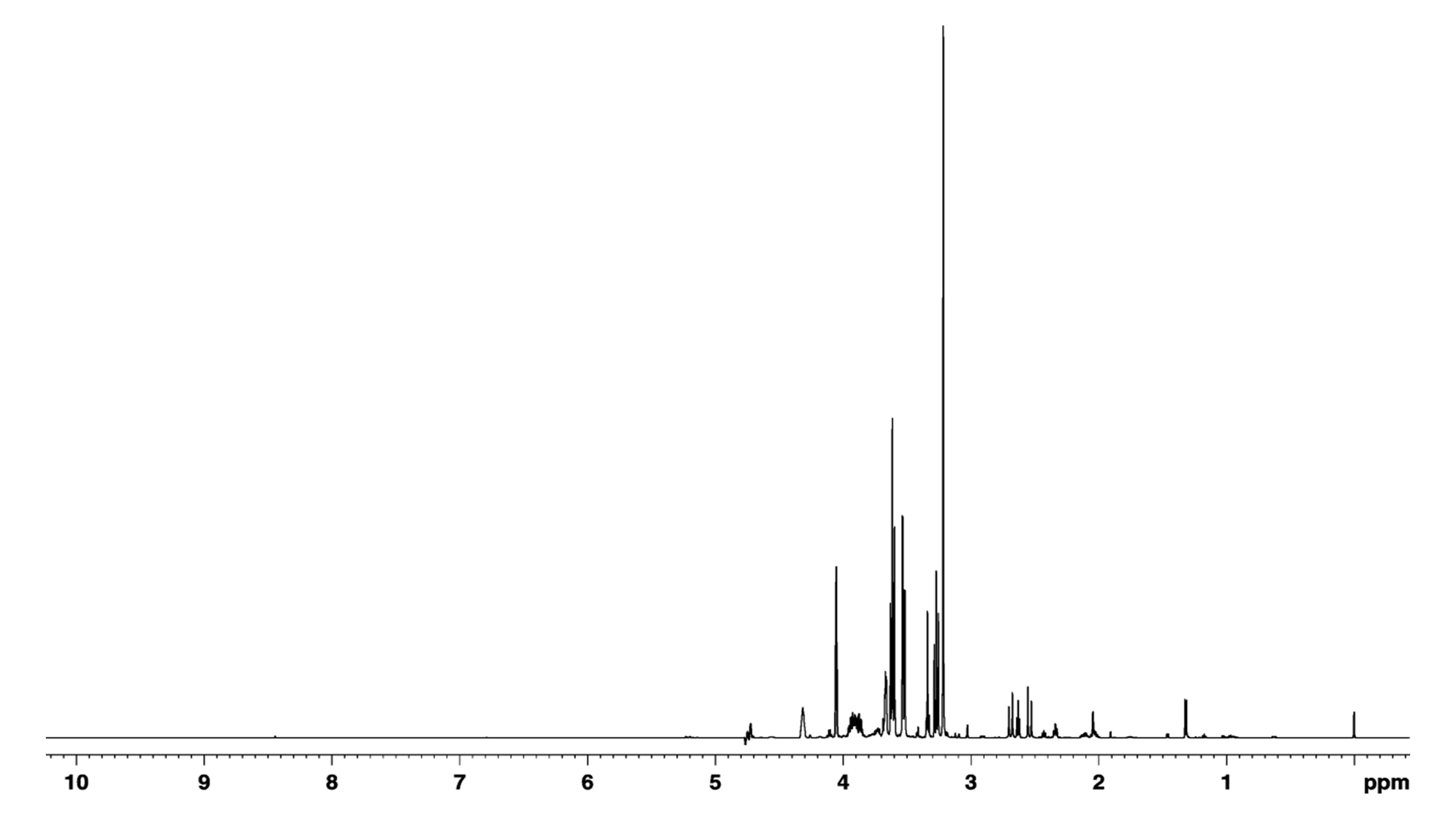

Supplement: Supplementary file 1 — Additional file 1 Supplementary Fig. 1 H-NMR (noesygppr1d) profile (600 MHz) from 0 to 8 ppm of pig seminal plasma. [file 40104_2021_636_MOESM1_ESM.tif]

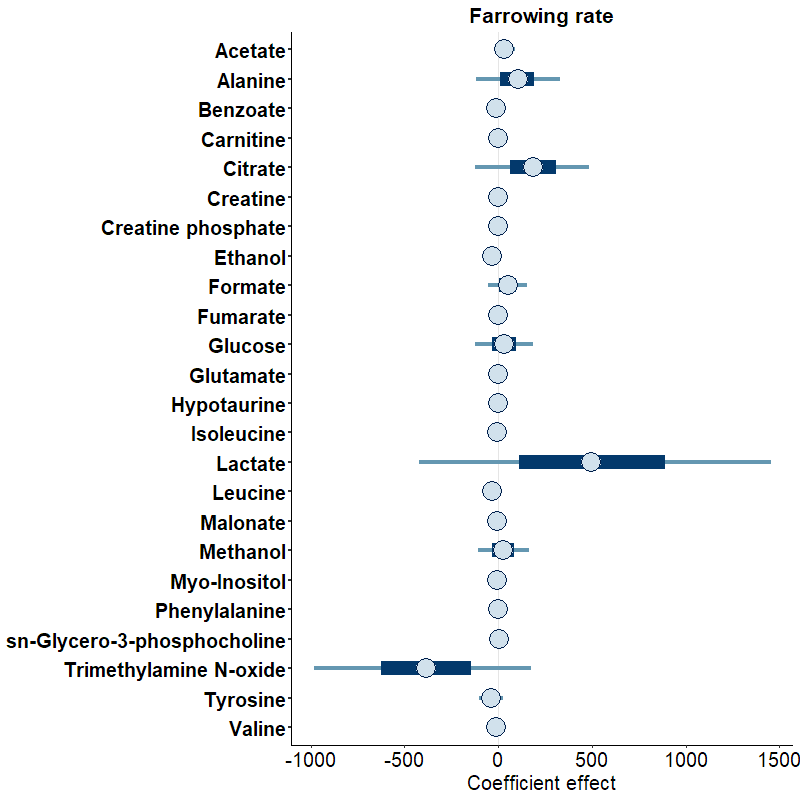

Supplement: Supplementary file 2 — Additional file 2 Supplementary Fig. 2 (A-D). Bayesian multiple logistic regression models for all the in vivo fertility parameters. The distribution of the coefficients (X axis) is depicted for each metabolite (Y axis). The coefficient distributions depict their effect on the model, as well as their associated uncertainties (credible intervals). Thus, changes in one unit on the coefficient value has a multiplicative effect on the log-odds of the prediction, equal to the value of the coefficient. Blue lines represent the 95% credible intervals, boxes show the 50% credible intervals, and dots are the distribution median. [file 40104_2021_636_MOESM2_ESM.zip › Supplementary Figure 2A_corrected.tiff]

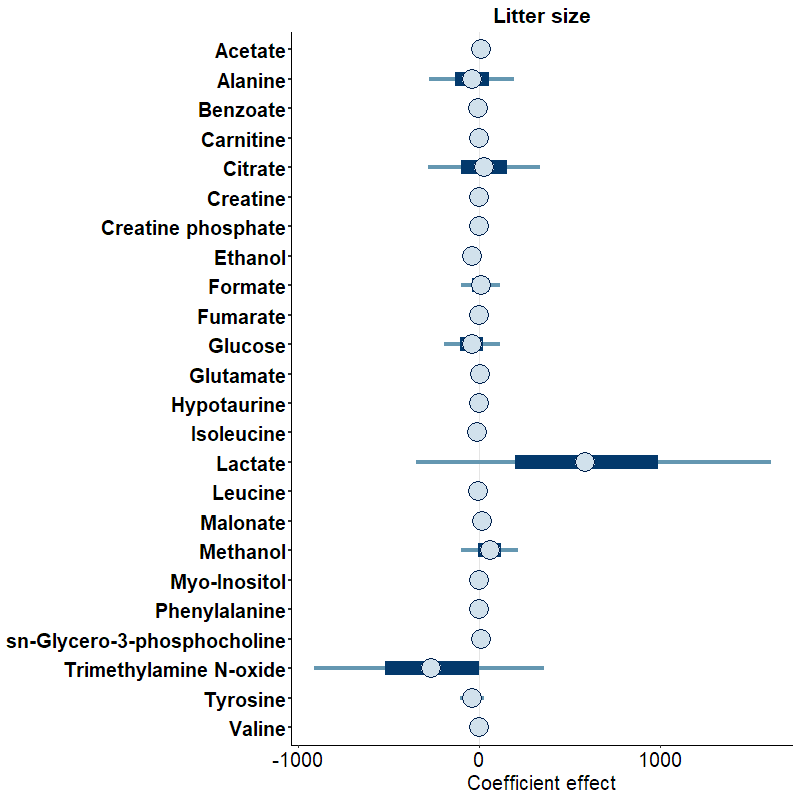

Supplement: Supplementary file 2 — Additional file 2 Supplementary Fig. 2 (A-D). Bayesian multiple logistic regression models for all the in vivo fertility parameters. The distribution of the coefficients (X axis) is depicted for each metabolite (Y axis). The coefficient distributions depict their effect on the model, as well as their associated uncertainties (credible intervals). Thus, changes in one unit on the coefficient value has a multiplicative effect on the log-odds of the prediction, equal to the value of the coefficient. Blue lines represent the 95% credible intervals, boxes show the 50% credible intervals, and dots are the distribution median. [file 40104_2021_636_MOESM2_ESM.zip › Supplementary Figure 2B_corrected.tiff]

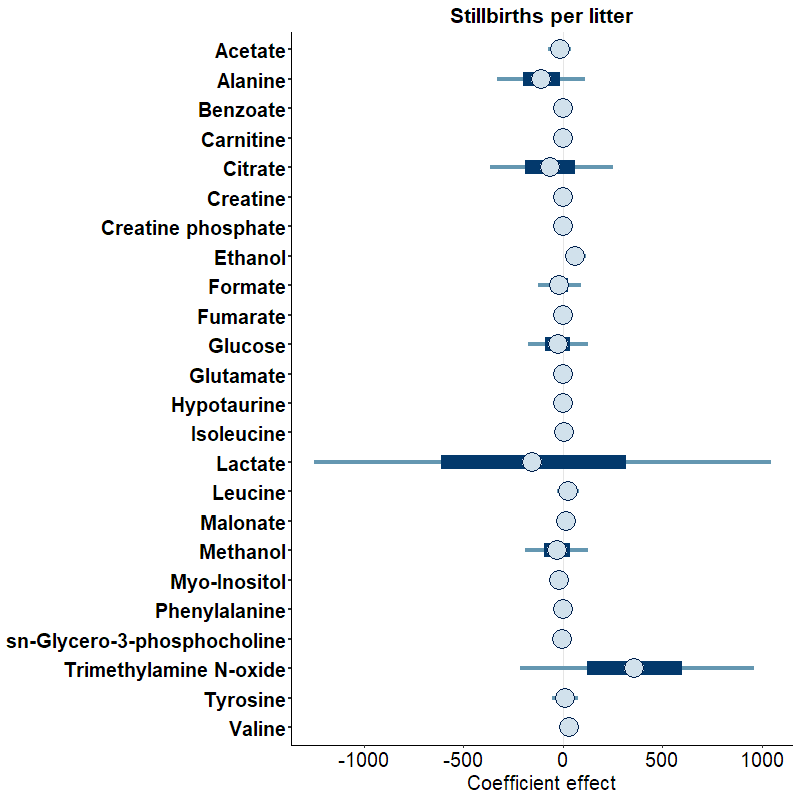

Supplement: Supplementary file 2 — Additional file 2 Supplementary Fig. 2 (A-D). Bayesian multiple logistic regression models for all the in vivo fertility parameters. The distribution of the coefficients (X axis) is depicted for each metabolite (Y axis). The coefficient distributions depict their effect on the model, as well as their associated uncertainties (credible intervals). Thus, changes in one unit on the coefficient value has a multiplicative effect on the log-odds of the prediction, equal to the value of the coefficient. Blue lines represent the 95% credible intervals, boxes show the 50% credible intervals, and dots are the distribution median. [file 40104_2021_636_MOESM2_ESM.zip › Supplementary Figure 2C_corrected.tiff]

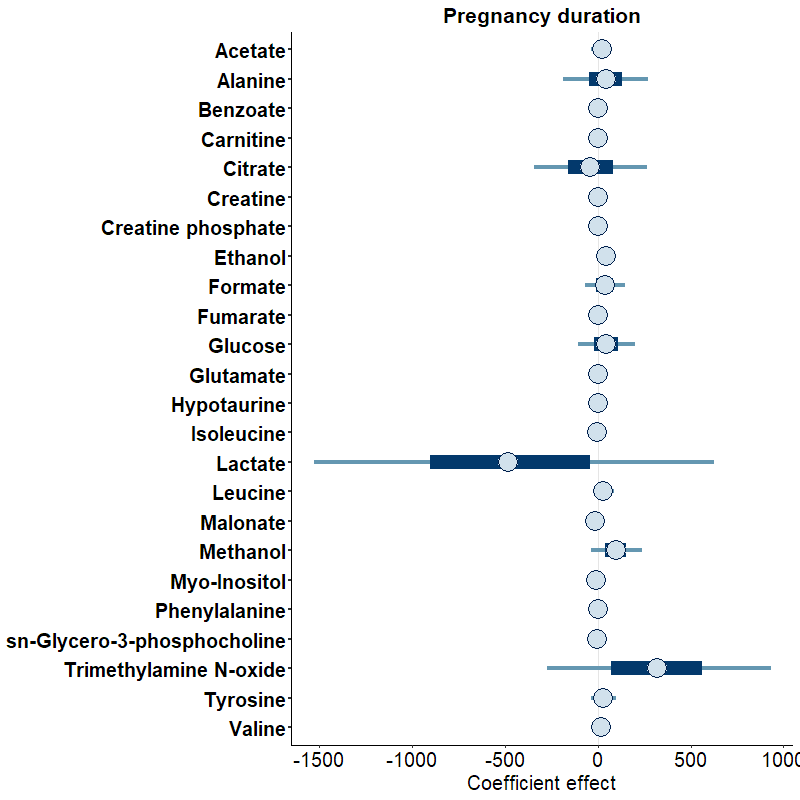

Supplement: Supplementary file 2 — Additional file 2 Supplementary Fig. 2 (A-D). Bayesian multiple logistic regression models for all the in vivo fertility parameters. The distribution of the coefficients (X axis) is depicted for each metabolite (Y axis). The coefficient distributions depict their effect on the model, as well as their associated uncertainties (credible intervals). Thus, changes in one unit on the coefficient value has a multiplicative effect on the log-odds of the prediction, equal to the value of the coefficient. Blue lines represent the 95% credible intervals, boxes show the 50% credible intervals, and dots are the distribution median. [file 40104_2021_636_MOESM2_ESM.zip › Supplementary Figure 2D_corrected.tiff]
